# Application of case-based study promoting the outcome-based education concept in clinical pharmacotherapeutic teaching: a pre-post study with implications for medical educator development

**DOI:** 10.3389/fmed.2026.1804644

**Published:** 2026-06-11

**Authors:** Meiling Yu, Shuying Dong, Daohua Guo, Yingying Huang, Lingti Kong, Xuhui Tong

**Affiliations:** 1 Faculty of pharmacy, Bengbu Medical University, Bengbu, Anhui, China; 2 Department of Pharmacy, The First Affiliated Hospital of Bengbu Medical University, Bengbu, Anhui, China

**Keywords:** case-based study, clinical pharmacotherapeutics, clinical teaching, outcome-based education, teaching effectiveness

## Abstract

**Background:**

Although outcome-based education (OBE) and case-based study (CBS) are commonly used in health professions education, there are ongoing issues with the standardization of faculty evaluation rubrics and the integration of teaching with core competencies. This study aimed to rectify these issues in clinical pharmacotherapeutic education.

**Methods:**

A total of 78 undergraduate clinical pharmacy students participated in this pre–post study. Teaching effectiveness was assessed using a novel, standardized OBE scoring rubric for teacher evaluations, alongside student self-assessments of engagement.

**Results:**

Significant improvements were observed between the first case study session and the final case study session across four main aspects in this study (*p* < 0.001): case reporting, PPT creation and presentation, question answering, and citation ability. Students’ self-scores confirmed significantly higher learning initiative and participation in the OBE-based CBS practical segment compared to the traditional theoretical teaching segment (*p* < 0.001). Notably, course interest increased significantly to 92.31% (vs. 74.36%, *p* = 0.003). These results showed that the standardized rubric effectively translated abstract OBE concepts into measurable teaching behaviors.

**Conclusion:**

The OBE-based CBS model, supported by a validated scoring system, effectively bridging the gap between knowledge and practice. This framework offers medical educators a replicable tool for standardizing curricula and monitoring quality.

## Introduction

As a core course in clinical pharmacy, clinical pharmacotherapeutics facilitates early learning in pharmacology, clinical diagnostics, internal medicine, and related disciplines. It provides the foundational knowledge of drug therapy and rational medication use, playing a critical role in developing students’ competencies in pharmaceutical care (1–4). The course is both knowledge-intensive and broad in scope, integrating theoretical concepts with strong practical and clinical relevance.

The course emphasizes the integration of theoretical knowledge with practical application. The primary objective of clinical pharmacotherapeutics is to provide students with a comprehensive understanding of the key principles of drug therapy, including adverse drug reactions (ADRs), drug interactions, the impact of disease states on medication management, and special considerations for specific patient populations. Students are also expected to master treatment strategies for common diseases and develop the ability to offer appropriate medication guidance to patients. However, due to the complexity of the subject, achieving these learning objectives through theoretical instructions alone is challenging. To better achieve the course objectives and develop practical clinical pharmacy skills, case-based study (CBS) teaching was conducted in the following theoretical sessions. Issues identified through student and teacher feedback during the initial phase of CBS teaching have been addressed. These included the absence of a standardized scoring system, excessive case study sessions, extremely difficult or numerous questions posed by some teachers, and limited interactivity during instruction. Following a discussion, we have adopted, for the first time, an outcome-based education (OBE) concept to the above CBS teaching in the practical sessions of this course. OBE is an advanced educational concept based on learning results, which is student-oriented and emphasizes their central position in teaching and learning (5–7).

A fundamental principle of the OBE framework is the constructive alignment among curriculum, teaching, and assessment (8, 9). Reports have indicated that, for an educational intervention to be effective, the curriculum design should articulate clear learning outcomes, teaching activities should facilitate the achievement of these outcomes, and assessment tasks should accurately evaluate them (10–12). This triad ensures that students are not simply exposed to content; rather, they are actively advancing toward competency.

According to the above teaching philosophy, the teaching goals are categorized into knowledge, capability, and quality goals in this study. The knowledge goal involves understanding pharmacotherapy regimens for common diseases, ADRs, drug interactions, the effects of diseases on clinical medication use, and medication considerations for special populations. The capability goal focuses on developing and evaluating pharmacotherapy regimens, conducting medication consultations, providing medication guidance, applying theoretical knowledge in practice, and improving the ability to communicate professional information. The quality goal aims to develop students’ ability to acquire new pharmacotherapy knowledge and stimulate their interest in scientific research. The course’s OBE objectives were achieved through CBS teaching. Designing case-based questions further enhances students’ proficiency in resolving clinical medication issues, guiding rational drug use, and ensuring medication safety. Guided by cases, CBS enables students to discuss case studies and actively seek solutions (13–17).

Despite the widespread adoption of OBE in health professions education, several gaps persist in the current literature (12, 18–22). First, although studies examine student academic performance, few have systematically assessed the alignment between specific teaching activities and the attainment of distinct competency goals, including knowledge, capability, and quality, within clinical pharmacotherapeutics. Second, there is a lack of empirical research on standardizing teacher evaluation rubrics to ensure consistent implementation of OBE, which is a crucial component of medical educator development.

To address these gaps, this study advanced beyond simple curriculum implementation to establish a closed-loop system of “objectives-teaching-assessment.” We aimed to address the following research inquiries: (1) Does the amalgamation of OBE and CBS markedly enhance student performance in terms of knowledge, capability, and quality goals? (2) What impact does the OBE-CBS model have on students’ subjective learning experiences, including learning initiative, course participation, satisfaction, and interest?

We hypothesized that students in the final session would achieve significantly higher scores in teacher evaluations (specifically in case reporting, PPT creation and presentation, question answering, and citation ability) compared to their performance in the first session. Furthermore, we anticipated that students’ self-assessed learning initiative, course participation, course satisfaction, and interest would be significantly greater in the OBE-based CBS practical segment than in the traditional theoretical segment. Therefore, teaching effectiveness was evaluated through both teacher evaluations and student self-assessments in this study.

## Methods

### Study participants

This study involved 78 undergraduate clinical pharmacy students from Bengbu Medical University, including 40 from the class of 2020 and 38 from the class of 2021. The clinical pharmacotherapeutic course comprised 84 class hours of theoretical instruction and 60 class hours of case-based learning. The study protocol was reviewed and approved by Bengbu Medical University (Bengbu, China) and written informed consent was obtained from each teacher and the students’ legal guardians or next of kin. All protocols were approved and were conducted in accordance with the school’s guidelines and regulations.

### Study methods

**Figure 1:**
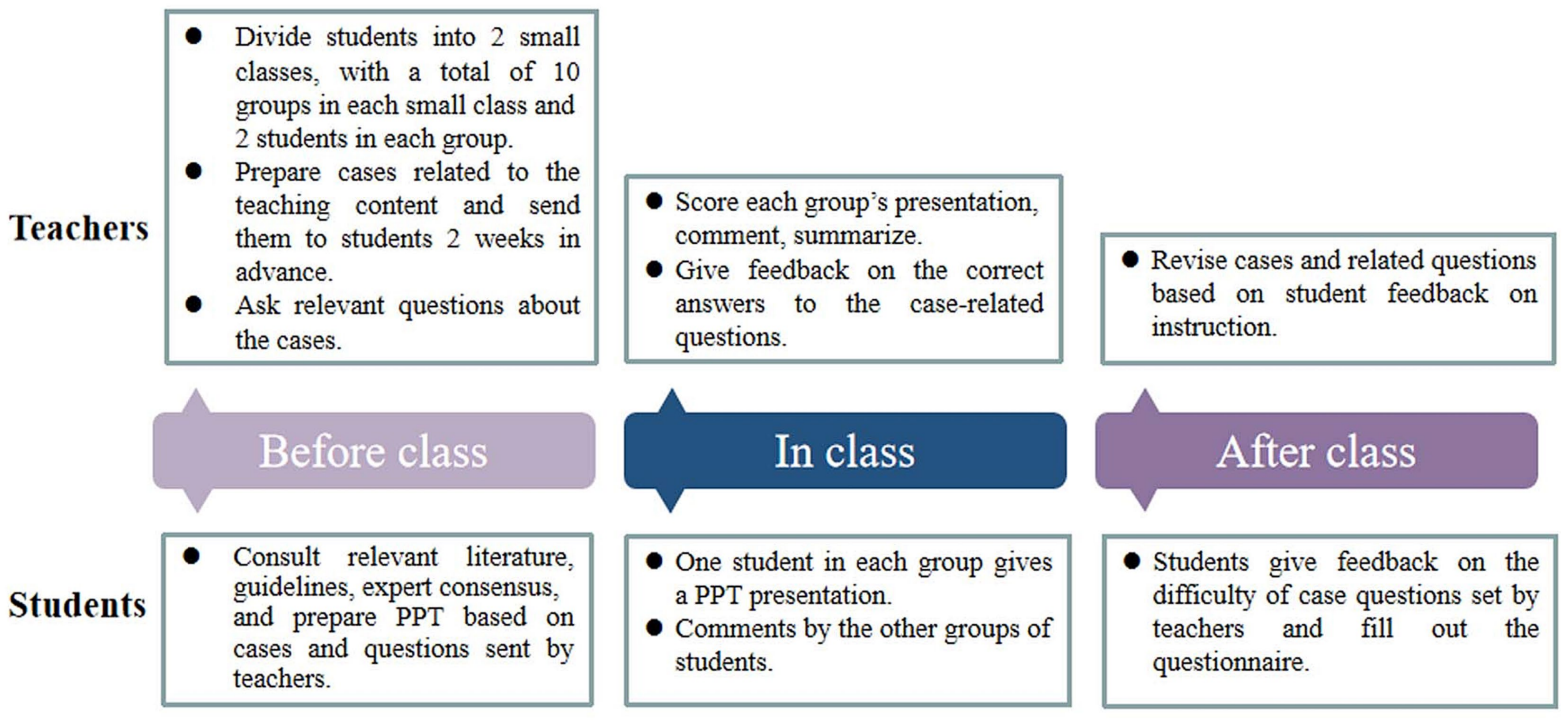
Teaching process of CBS based on the OBE concept.

Students undertook CBS courses after completing the corresponding theoretical chapters; the teaching process is illustrated in Figure 1, and a total of 12 cases were studied.

Based on the OBE concept, the 12 cases were designed to achieve three objectives: (1) Knowledge goal—students gain mastery of pharmacotherapy regimens for common diseases; understand ADRs, drug interactions, and the impact of diseases on medication use; and understand pharmacotherapy for special populations. (2) Capability goal—students learn to develop and evaluate pharmacotherapy regimens, provide medication consultations and guidance, apply clinical knowledge, and communicate professionally. (3) Quality goal—students are encouraged to pursue new pharmacotherapy knowledge and develop an interest in scientific research. Each case included 3–5 guiding questions to support the achievement of these objectives.

The complexity of the cases and the associated questions was evenly distributed among the groups to prevent any case from being intentionally simplistic or extremely complex. During the lesson preparation, all participating teachers engaged in comprehensive discussions regarding the 12 cases and their respective questions; subsequently, the teaching staff revised both the cases and the questions based on their collective insights, and the difficulty and complexity of the cases are intentionally maintained at a consistent level.

**Figure 2:**
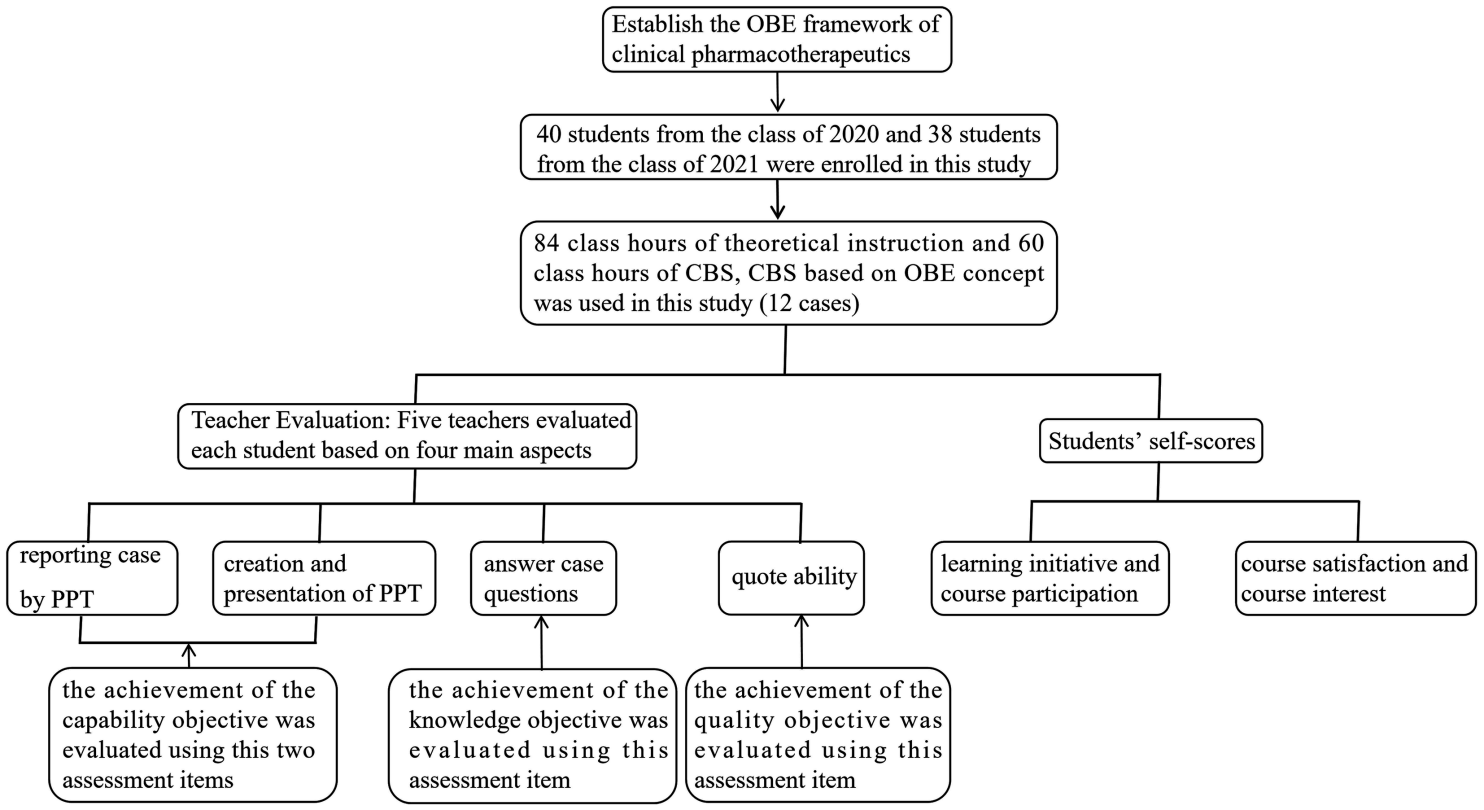
The schematic diagram of this study.

The study schematic diagram is illustrated in Figure 2, and the teaching effectiveness was assessed through teacher evaluations and student self-assessments.

### Teaching effect evaluation

#### Teacher evaluation

Five teachers evaluated each student based on four main aspects (Table 1); the final score for each student was calculated as the average of the five teachers’ scores.

**Table 1:** Scoring table of the case course and the OBE evaluation indicators.

Items of teacher evaluation		Score	Goals of OBE concept	Score of each group
Reporting case by PPT	Describe the patient’s general information correctly	6	Capability goal	
	Describe the progression of the disease clearly	6		
	Analyze the clinical significance of related diagnostic indicators appropriately	6		
	Summarize and evaluate the main issues in the drug treatment plan correctly and analyze the rationale and shortcomings of the pharmacotherapy regimens correctly	6		
	Offer medication guidance to the patient correctly	6		
Creation and presentation of PPT	Well-designed PPT slides and good layout	10		
	Fluent PPT presentation and familiarity with the content	10		
	Completeness of the PPT	10		
Answer case questions	Answer case questions correctly	30	Knowledge goal	
Quote ability	Quote high-quality references, expert consensus, and guidelines to answer case questions	10	Quality goal	
Total score		100		

The first evaluation item, worth 30 points, was a case report presented via a PowerPoint presentation. Students were required to (a) accurately describe the patient’s general information (e.g., gender, age, medical history, past history, family history, and diagnosis), (b) clearly explain the disease progression, (c) appropriately analyze the clinical significance of relevant diagnostic indicators, (d) correctly summarize and assess the key issues in the pharmacotherapy plan, (e) evaluate the rationale and limitations of the treatment regimen, and (f) provide appropriate medication guidance for the patient.

The second evaluation item, worth 30 points, assessed the creation and presentation of PPT slides. The evaluation criteria included slide design, layout, presentation fluency, familiarity with the content, and overall completeness. The content of the PPT was required to include case information, responses to case questions, a summary, and the cited references, expert consensus, or guidelines.

The third item, worth 30 points, evaluated the relevance and accuracy of responses to case questions.

The fourth item, worth 10 points, assessed the ability to cite high-quality references, expert consensus statements, and clinical guidelines in answering case questions.

To evaluate the effectiveness of this teaching mode, student performance in the four assessment areas was compared between the first and last practical sessions in this study to determine any improvements. The final score for each student was calculated as the average of the five teachers’ evaluations; this scoring system served as a preliminary assessment of the achievement of OBE objectives.

(a) Knowledge goal: understanding pharmacotherapy regimens for common diseases, recognizing ADRs, drug interactions, and the impact of diseases on medication use, and comprehending pharmacotherapy considerations for special populations are all knowledge goals. The achievement of the knowledge objective was evaluated using the third assessment item, which measured students’ ability to accurately answer case-based questions provided by teachers.
(b) Capability goal: developing and evaluating pharmacotherapy regimens for common diseases, conducting medication consultations and providing appropriate guidance, applying clinical knowledge in practice, and enhancing the ability to communicate professional information effectively are all capability goals. The achievement of the capability objective was assessed using the first and second evaluation items; these items involved case reporting, analysis of pharmacotherapy regimens during presentations, provision of medication guidance to patients, and assessment of PPT design and delivery.
(c) Quality goal: fostering students’ ability to acquire new pharmacotherapy knowledge and stimulating their interest in scientific research are the quality goals. The achievement of the quality objective was assessed using the fourth evaluation item, which examined students’ ability to effectively cite high-quality references, expert consensus statements, and clinical guidelines in responding to case questions.

#### Students’ self-scores for learning initiative and course participation

Following the CBS course, all 78 students self-assessed their learning initiative and participation in both the traditional theoretical teaching segment and the OBE-based CBS practical segment, with each item scored on a 10-point scale: 1–3 points indicating poor learning initiative; 4–7 points indicating average learning initiative; and 8–10 points indicating relatively high learning initiative and 1–3 points indicating low course participation; 4–7 points indicating average course participation; and 8–10 points, relatively high course participation. Additionally, changes in students’ self-evaluated scores for learning initiative and course participation were compared between the traditional theoretical teaching segment and the OBE-based CBS practical segment.

#### Evaluation of course satisfaction and interest

Course satisfaction was classified into three levels: satisfied, generally satisfied, and dissatisfied. Similarly, course interest was divided into three categories: interesting, generally interesting, and uninteresting. All 78 students participated in the evaluation of both course satisfaction and interest. The course satisfaction rate was calculated as follows: the number of students selecting “satisfied” + “generally satisfied”/total number of students × 100%, and the course interest rate was calculated as follows: the number of students selecting “interesting” + “generally interesting”/the total number of students × 100%.

### Statistical methods

SPSS 22.0 was used for statistical analysis, and the measurement data were expressed as $\bar{X}$ ± *s*. The independent sample *t*-test or paired *t*-test was used for the comparison of normally distributed data. The Mann–Whitney *U*-test or Wilcoxon signed-rank test was conducted to compare the two groups with not normally distributed data (23, 24). Count data were expressed as a rate (%) and subjected to a chi-square test. *p*-values of <0.05 indicated a statistically significant difference.

## Results

### Changes in teacher scores from the first to the last case study session in this study

**Figure 3:**
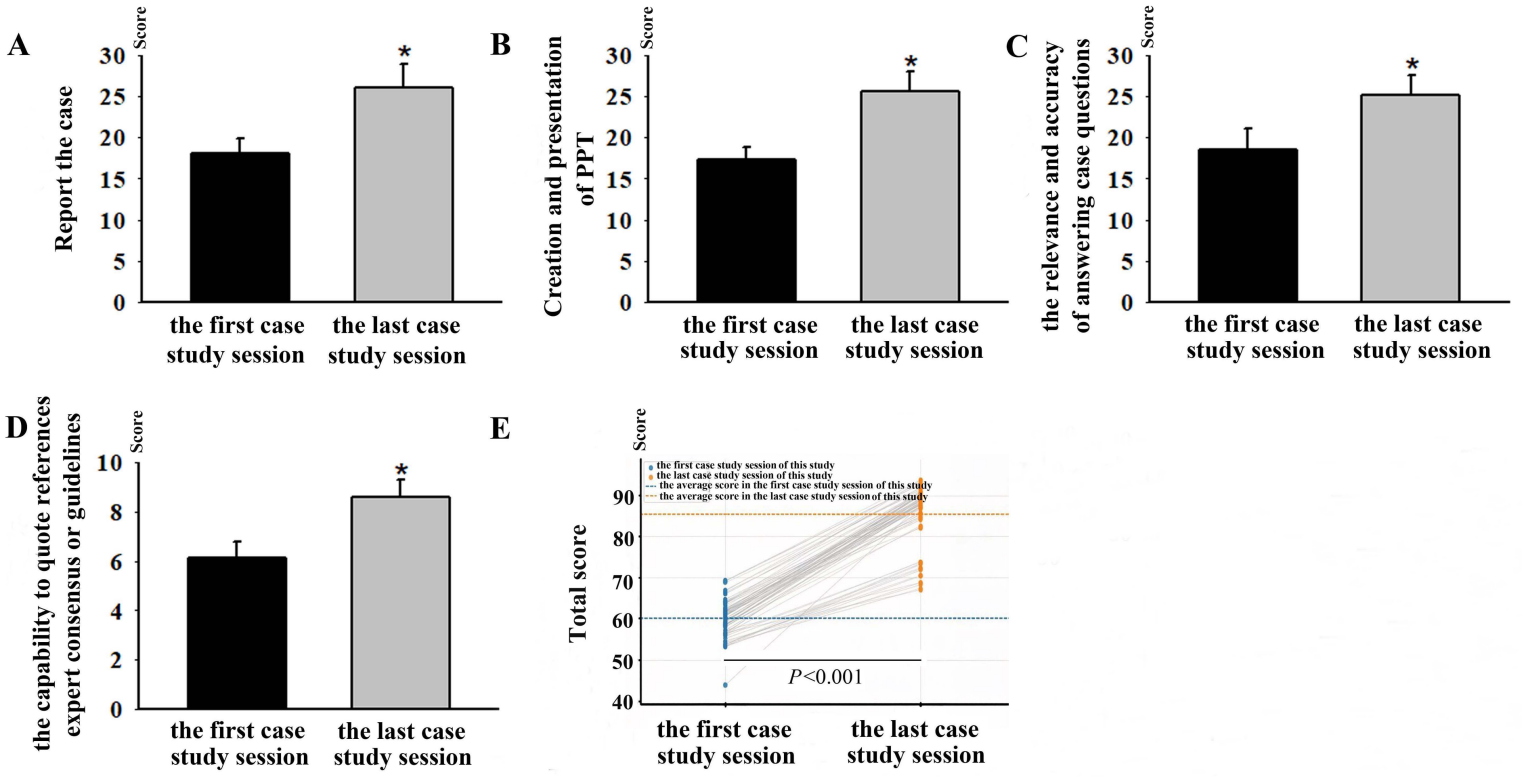
Scores of the first and last case study sessions. **(A)** Scores for case report. **(B)** Scores for the creation and presentation of PPT. **(C)** Score for the relevance and accuracy of answering case questions. **(D)** Score for the capability to quote high-quality references, expert consensus, or guidelines to answer case questions. **(E)** The total score for the 78 students in the first session and the last case study session.

During the first and last case study sessions in this study, 5 teachers evaluated 78 students across four aspects: case reporting, the creation and presentation of the PPT, the relevance and accuracy of responses to case questions, and the ability to cite high-quality references, expert consensus, or guidelines when answering questions. The results showed that scores in the final case study session were significantly higher than those in the initial session across all four aspects in this study: 26.02 ± 2.98 *vs.* 18.07 ± 1.84 (Figure 3A), 25.65 ± 2.31 vs. 17.39 ± 1.38 (Figure 3B), 25.16 ± 2.31 vs. 18.59 ± 2.54 (Figure 3C), and 8.61 ± 0.71 vs. 6.12 ± 0.69 (Figure 3D) (*p* < 0.001). The total score of the 78 students also increased significantly in the final session (85.44 ± 7.33) compared to the initial session (60.16 ± 4.41) (Figure 3E*, p* < 0.001).

### Students’ self-scores for learning initiative and course participation

**Figure 4:**
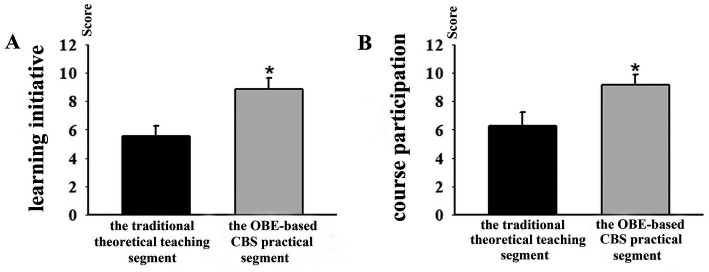
The students’ self-scores for learning initiative and course participation in the traditional theoretical teaching segment and OBE-based CBS practical segment. **(A)** The students’ self-scores for learning initiative in the traditional theoretical teaching segment and OBE-based CBS practical segment. **(B)** The students’ self-scores for course participation in the traditional theoretical teaching segment and OBE-based CBS practical segment.

The students’ self-scored learning initiative and course participation in the traditional theoretical teaching segment and the OBE-based CBS practical segment were observed. The results showed that the self-scores for learning initiative and course participation for the OBE-based CBS practical segment were 8.87 ± 0.78 and 9.14 ± 0.75 points (Figures 4A,B), respectively, which were significantly higher than those for the traditional theoretical teaching segment (5.53 ± 0.75 points and 6.26 ± 0.97 points, respectively, *p* < 0.001).

### Comparison of course satisfaction and course interest between the traditional theoretical teaching segment and the OBE-based CBS practical segment

A total of 78 students evaluated their satisfaction and interest in both the traditional theoretical teaching segment and the OBE-based CBS practical segment. Student satisfaction was 89.74% for the OBE-based CBS practical segment and 85.90% for the traditional theoretical teaching segment (Table 2), with no statistically significant difference (*p* = 0.463). Interest in the OBE-based CBS practical segment was 92.31%, which was significantly higher than in the traditional theoretical teaching segment (74.36%) (Table 3, *p* = 0.003). These findings indicate that the CBS course based on the OBE concept significantly enhanced students’ interest in clinical pharmacotherapeutics.

**Table 2:** Course satisfaction survey.

Grouping	Satisfied	Generally satisfied	Dissatisfied	Satisfaction	*χ*^2^ value	*p*-value
Traditional theoretical teaching segment	52	15	11	85.90%	0.539	0.463
OBE-based CBS practical segment	57	13	8	89.74%		

**Table 3:** Course interest survey.

Grouping	Interesting	Generally interesting	Uninteresting	Interest	*χ*^2^ value	*p*-value
Traditional theoretical teaching segment	38	20	20	74.36%	9.046	0.003
OBE-based CBS practical segment	58	14	6	92.31%		

## Discussion

Clinical pharmacotherapeutics is a core course in the undergraduate clinical pharmacy curriculum, aimed at preparing graduates to provide clinical pharmacy services. This study implemented CBS approaches within the framework of OBE; the results showed significant improvements in course satisfaction, student interest, teachers’ evaluations, and student self-assessments.

In this study, the “Case Report” section of the scoring rubric included criteria such as “a clear description of disease progression”, “appropriate analysis of the clinical significance of diagnostic indicators”, “evaluation of key issues in pharmacotherapy”, and “provision of appropriate medication guidance for patients”. These criteria required students not only to summarize the cases but also to engage in analysis, judgment, and decision-making. This process simulated the core workflow of clinical pharmacists. The quality of its execution directly reflected students’ foundational abilities to formulate and evaluate therapeutic plans and provide medication guidance. The “PPT Production and Presentation” section evaluated content completeness, production quality, and presentation fluency. This process required students to transform fragmented knowledge into structured and visually organized professional reports, which were then presented to their peers. This process encouraged students to integrate knowledge, organize their reasoning, and communicate their ideas effectively. This stage provided a direct demonstration of students’ ability to apply knowledge and communicate professionally; consequently, scores in these two areas reflected not only the quality of task completion but also measurable indicators of the targeted competencies. Improvements in these scores indicated that students had developed essential professional competencies through these practical training experiences.

Evaluating knowledge objectives required assessing students’ ability to identify, recall, and understand the core concepts of the course and to apply this knowledge accurately in specific contexts. The scoring criterion “relevance and accuracy of answers to case questions” directly assessed students’ ability to identify key elements—such as diseases, drugs, and special populations—from case information and to apply their knowledge appropriately in their responses. The degree of relevance indicated the appropriateness of knowledge application, whereas accuracy reflected the depth of students’ understanding of the core concepts; therefore, the score for this component provided a direct and objective measure of the extent to which students achieved the knowledge objectives. The quality objective emphasized the development of students’ potential attributes and interest in autonomous learning and scientific inquiry rather than the acquisition of specific knowledge or skills. In case-based teaching, requiring students to cite references, expert consensus statements, and clinical guidelines guides them in acquiring new knowledge about pharmacotherapy. Students should actively search for, screen, evaluate, and cite authoritative evidence to support their viewpoints, thereby moving beyond the passive acceptance of knowledge from textbooks or instructor-led teaching. This process simulated the fundamental steps of literature retrieval and evidence application in scientific inquiry. The ability to accurately cite authoritative evidence indicated that students were beginning to develop evidence-based thinking and to acquire methods for tracking developments in their professional fields; changes in scores reflecting this ability could measure students’ transition from learners to knowledge explorers and indicate whether their interest in scientific research had been stimulated. The practical course grading rubric used in this study aimed to translate outcome-based teaching objectives into observable and measurable behavioral indicators; thus, the significant improvement in case-teaching scores indicated enhanced knowledge acquisition, competency development, and quality cultivation among students.

Our findings were consistent with previous studies (25–27), indicating that the OBE-based teaching approach enables students to apply knowledge, broaden their knowledge base, develop innovative thinking, and develop a service-oriented mindset, thereby promoting continuous course reform. In contrast to prior studies on OBE (12, 18–22), which primarily focused on designing course outlines or relied heavily on summative assessments, this study established three comprehensive teaching objectives aligned with the core competencies required in clinical pharmacy; each objective was paired with specific classroom activities and corresponding assessment indicators, forming a closed loop of “objectives–teaching–assessment.” Furthermore, by tracking and comparing performance changes within the same group of students across identical assessment dimensions at the beginning and end of the teaching cycle, this study provided empirical evidence supporting the effectiveness of OBE-based teaching; it also emphasized stimulating and evaluating students’ evidence-based literacy and autonomous learning abilities, which are key quality objectives in case-based teaching. These objectives align with the principles of “continuous improvement” and “lifelong learning” within the OBE framework for pharmaceutical education. However, this study had several limitations, especially the limitations in measuring the effectiveness of the CBS on OBE; for example, the impact of the teaching reform on long-term indicators of professional development, such as clinical internship performance and post-graduation employability, was not tracked, limiting the understanding of its sustained effects. While CBS proved effective in assessing clinical reasoning (capability goal), it may not represent the most suitable approach for evaluating the breadth of factual knowledge (knowledge goal). Moreover, the study did not include a traditional lecture-based teaching group as a control. Therefore, the observed improvement may be partially attributed to students’ familiarity with the course format and cumulative knowledge acquisition rather than the specific effects of the OBE-CBS model. Future research should further optimize the study design in light of these limitations.

In addition to benefiting students, the findings of this study have important implications for the development of medical educators. The standardized scoring rubric functions not only as an assessment tool for students but also as a framework for faculty training. For medical educators, the translation of abstract OBE concepts into specific behavioral indicators, such as “appropriate analysis of diagnostic indicators” or “correct citation of guidelines”, frequently represents the most challenging aspect of curriculum reform. Our study offers a replicable model that enables educators to align their instructional design with core competencies, thereby facilitating a successful transition in the faculty role from knowledge transmitters to facilitators of evidence-based inquiry. Building on these findings, we have initiated institutional-level reforms. We have revised the syllabus for the clinical pharmacotherapy course. Additionally, a standardized revision of lesson plans and lecture notes has become essential, and all instructors are expected to incorporate the OBE concept into their teaching designs. These elements have been integrated into the teaching quality monitoring system to ensure the effective and sustainable implementation of the reform. This comprehensive approach highlights the significance of faculty development programs that emphasize “assessment literacy” and “evidence-based teaching strategies”.

This study establishes a foundation for the implementation of OBE; however, future research should use more rigorous mixed-methods designs to further investigate its effects on medical educators. First, quantitative measures, including the development and validation of an “OBE Teaching Competency Scale”, should be used to evaluate changes in teachers’ instructional skills and metacognitive awareness. Second, the quantitative data should be triangulated with qualitative data obtained from semi-structured interviews and reflective teaching journals. These interviews should aim to capture the conceptual shifts in educators’ beliefs about student-centered learning and the challenges encountered during curriculum implementation. Finally, longitudinal studies that track teachers’ professional growth trajectories in conjunction with student outcomes would offer a comprehensive perspective on the integration of OBE in clinical pharmacy education.

## Conclusion

CBS teaching based on the OBE concept can effectively support the achievement of the course’s knowledge, capability, and quality goals in clinical pharmacotherapeutics; this OBE-based teaching approach shows promising potential for broader implementation. In this future, we will enhance the OBE evaluation system based on this research, aiming to provide additional guidance for the teaching reform associated with the OBE concept in our institution.

## Data Availability

The original contributions presented in the study are included in the article/supplementary material, further inquiries can be directed to the corresponding authors.
